# Dose tranexamic acid reduce blood loss associated with simultaneous bilateral distal tibial tubercle-high tibial osteotomy?

**DOI:** 10.1186/s12891-021-04831-3

**Published:** 2021-11-10

**Authors:** Zhimeng Wang, Qiang Huang, Lu Liu, Yao Lu, Congming Zhang, Teng Ma, Zhong Li, Qian Wang, Hanzhong Xue, Kun Zhang

**Affiliations:** Department of Orthopaedics and Trauma, Hong Hui Hospital, Xi’an Jiaotong University, No. 555, East Youyi Road, Xi’an, 710000 Shaanxi China

**Keywords:** Tranexamic acid, Blood loss, High tibial osteotomy, Simultaneous bilateral

## Abstract

**Background:**

Simultaneous bilateral distal tibial tubercle high tibial osteotomy (SBDTT-HTO) can result in increased blood loss. The aim of this study is to evaluate the actual hemostatic effect of different tranexamic acid (TXA) treatment regimen in SBDTT-HTO.

**Methods:**

We conducted a retrospective case–control study including 54 patients who underwent SBDTT-HTO. The single-dose group (*n* = 18) received 1 g of intravenous TXA 15–30 min before surgery, the two-dose group (*n* = 18) received an additional 1 g of intravenous TXA 6 h after surgery, and the multiple-dose group (n = 18) received an additional 1 g intravenous TXA per-day until discharge. Blood loss, hemoglobin levels, occurrence of any adverse events,functional analysis, quality of life, and pain assessmentswere compared among the three groups.

**Results:**

The total blood loss, hidden blood loss, drainage volumes, and haemoglobin level in the multiple-dose group all occupy a significant advantage.(*p* < 0.05). In addition, better quality of life were observed in patients belonging to the multiple-dose group then single-dose group.(*p <* 0.05).

**Conclusions:**

Based on our results, for patients undergoing SBDTT-HTO, sequential intravenous TXA administration can effectively and safely reduce blood loss,maintain postoperative Hb levels,and with the advantage of accelerating recovery.

## Background

Knee osteoarthritis (KOA) is a frequently occurring disease in elderly patients [1]. With continuous advances in clinical and basic research, the step treatment plan, implemented by a majority of doctors and widely accepted by patients, along with high tibial osteotomy (HTO) is garnering interest [2–4]. Compared with the traditional closing-wedge HTO (CW-HTO), the distal tibial tubercle HTO (DTT-HTO) is associated with low adverse events and high survival rates. Hence, it is considered one of the most effective methods for the treatment of KOA [5–7].

However, owing to the use of tourniquets, exfoliation of soft tissues, and exposure of the cancellous bone surface during osteotomy, intraoperative and postoperative bleeding is inevitable and may lead to significant blood loss [8]. Postoperative bleeding can cause haematomas around the incision, delayed incision healing, deep infections, and anaemia [9]. Patients with massive blood loss require allogeneic blood transfusion, which can lead to adverse reactions such as fever, infection, allergic reactions, and haemolysis [10]. Therefore, blood management associated with DTT-HTO is an important and widely discussed topic for surgeons. Blood management for patients undergoing simultaneous bilateral DTT-HTO (SBDTT-HTO) is more challenging than that for patients undergoing single DTT-HTO.

As an important part of blood management, anti-fibrinolytic therapy has attracted the attention of researchers and clinicians. Tranexamic acid (TXA), a traditional anti-fibrinolytic drug, can effectively reduce the perioperative blood transfusion rate and dominant and hidden blood loss (HBL) and does not increase the risk of deep vein thrombosis (DVT). Hence, it has become the gold standard for perioperative blood management [11, 12]. Previous studies have confirmed that intravenous (IV) TXA administration in simultaneous bilateral total knee arthroplasty (TKA) can effectively reduce total blood loss (TBL) and allogeneic blood transfusion needs, without any additional thromboembolic risk [13–15].

However, to date, there are no relevant reports evaluating the efficacy of different IV TXA in SBDTT-HTO. Thus, a retrospective case–control study was conducted at our institution to answer the following questions: (1) can sequential IV TXA further reduce perioperative blood loss and postoperative drainage volume; (2) does sequential IV TXA have any additional advantages in functional recovery, quality of life (QoL), and pain reduction during postoperative rehabilitation; and (3) does the administration of sequential IV TXA increase the occurrence of thromboembolic events?

## Materials and methods

### Patients

This study retrospectively analysed the clinical data from patients who underwent SBDTT-HTO between January 2017 and December 2020. SBDTT-HTO is performed under a single dose of anaesthesia. The inclusion criteria were as follows: (1) patients with symptomatic medial osteoarthritis, (2) age 40–60 years, (3) no coagulopathy and abnormal haemoglobin (Hb) levels prior to the operation. The exclusion criteria were as follows: (1) patients who underwent staged DTT-HTO for bilateral KOA; (2) those with severe brain, heart, liver, and kidney dysfunction who could not tolerate surgery; (3) those with blood system diseases; (4) post-traumatic knee arthritis; (4) those undergoing bilateral UKA surgery; (5) those with congenital or acquired clotting disorder, a history of DVT or pulmonary embolism (PE), cardiovascular problems, or a known allergy to TXA; and (6) those with incomplete medical data/history.

### Study design and blood management

Based on the established inclusion and exclusion criteria, 54 patients were enrolled in the study, with 18 patients each assigned to the single-dose, two-dose, and multiple-dose groups. The single-dose group was administered 1 g of IV TXA 15–30 min before the operation. The two-dose group received an additional 1 g of IV TXA 6 h after surgery. The multiple-dose group received an additional 1 g of IV TXA per-day until the third postoperative day (POD#3). The RBC transfusion indications formulated by our institution were as follows: (1) Hb < 70 g/L and (2) 70 g/L < Hb < 100 g/L,when patients has tachycardia, pallor and lethargy, poor appetite, and fatigue. Considering several studies have confirmed that a single dose of IV TXA (1 g) reduces TBL compared with that in the control group (normal saline) in major orthopedic surgery, the present study did not include a control group.

### Surgical methods and postoperative management

The operations were performed by the same group of experienced physicians, and the drugs that were administered under general anaesthesia were common among the three groups. A pneumatic tourniquet was used in all patients and was inflated to 100 to 120 mmHg higher than the systolic blood pressure,and the mean arterial blood pressure was maintained within 60–70 mmHg. Before osteotomy, an arthroscopic examination was performed to evaluate the cartilage of the medial and lateral compartments and the patellofemoral joint. Arthroscopic debridement, including meniscectomy or synovectomy, was performed, if necessary. DTT-HTO was then performed. A 5-cm longitudinal incision was made on the medial side of the knee joint to loosen the superficial layer of the medial collateral ligament. The C-arm machine fluoroscopically guided the position and direction of osteotomy with the Kirschner wire; the lateral hinge was located at the level of the upper tibiofibular joint. The angle between the Kirschner wire guide and the line connecting the tips of the two femoral condyles (horizontal line of the tibial plateau) was 30°. The osteotomy line was made in the direction of the Kirschner wire guide. In the lateral part of the intact tibia, five holes were drilled with 2.8-mm Kirschner wires to decrease the stress on the lateral cortical bone. A bony hinge 1-cm lateral to the knee was constructed using a matching osteotomy orthopaedic tool to slowly open it to avoid lateral hinge fracture, and the intersecting angle between the femoral condyles and the fibula axis was adjusted to 93°. Finally, the fixation of osteotomy was completed using a π-plate and locking screws.

All patients received drains, which were removed when the volume of the drain was less than 30 mL/24 h. Functional exercise of the ankle including active and passive range of motion was started on the first postoperative day (POD#1), and knee flexion–extension exercises and straight-leg raise exercises were conducted under the guidance of a physician on the second postoperative day (POD#2). Partial weight-bearing exercises were performed at 1–4 weeks, and full weight-bearing exercises were performed at 6–8 weeks postoperatively under the guidance of a physician. Antibiotics (ceftazidime, IV 2.0 g BID) were used as a preventive measure during the operation and on POD#1. All patients were treated with anticoagulants (enoxaparin, SC 20 mg qd) and intermittent compression boots as a preventive treatment for lower extremity venous thrombosis during hospitalisation and then treated with oral anticoagulants (rivaroxaban, Oral 10 mg qd) up to 35 days after discharge. Doppler ultrasound examinations were performed daily to detect DVT during hospitalisation.

### Outcome measurements

#### Primary outcomes

Perioperative TBL, HBL, and transfusion rate were the primary outcomes measured in this study. TBL was calculated by applying the Gross [16] and Nadler [17] formulas as follows:

$$\mathrm{PBV}\ \left(\mathrm{L}\right)={\mathrm{K}}_1\times {\mathrm{h}}^3+{\mathrm{K}}_2\times \mathrm{w}+{\mathrm{K}}_3$$

[h: height (m); w: weight (kg); for male patients, K_1_ = 0.366 9, K_2_ = 0.032 19, K_3_ = 0.604 1; for female patients, K_1_ = 0.356 1, K_2_ = 0.033 08, K_3_ = 0.183319]

$$\mathrm{TBL}\ \left(\mathrm{mL}\right)=\mathrm{PBV}\times \left({\mathrm{Hct}}_1-{\mathrm{Hct}}_2\right)+{\mathrm{Hb}}_{\mathrm{trans}}$$

[Hct_1_ was the first routine blood test after the patient was admitted to the hospital; Hct_2_ was the lowest postoperative value obtained by routine blood tests; and Hbtrans is the weight of the transfused packed red blood cells (PRBCs), where two units of PRBCs can cause an Hb increase of approximately 5.2 g/dL, with a volume of approximately 400 mL].

#### Secondary outcomes

Data on the duration of surgery and hospitalisation, drainage volume, and maximum Hb drop were also included in the statistical analysis. Furthermore, routine blood tests and coagulation tests (such as Hb, Hct, D-dimer, FDP,) were performed on POD#1 and POD#3. To evaluate the safety of TXA in this study, the occurrence of any vascular event within 12 weeks of surgery, including DVT of the lower extremity confirmed by ultrasound or PE confirmed by pulmonary spiral CT, was examined. The incidence of wound complications, such as dehiscence, haematoma, edge necrosis, and infection, and the potential adverse effects of TXA, such as epilepsy, rash, headache, nausea, and vomiting, were also recorded [18].

#### Knee function, QoL, and pain analysis

Preoperative knee function, QoL, and pain were assessed using the Lysholm knee score (LKS), 12-item Short Form Health Survey (SF-12), and visual analogue scale (VAS) of pain, respectively. LKS, SF-12, and VAS pain tests were repeated at 6- and 12-weeks postoperatively during routine outpatient visits. The VAS pain test was further assessed on POD#2 and POD#4.

### Statistical analysis

Statistical analyses were performed using GraphPad Prism 8.0 and SPSS version 22.0. Continuous variables are reported as the mean ± standard deviation. One-way analysis of variance was used to compare the differences among multiple groups and LSD-t post-hoc tests was used to compare the differences between two groups. While the Kruskal–Wallis H test and Mann–Whitney U test were used for nonparametric data. Chi-square test and the Fisher exact test were used to analyze the qualitative variables. Statistical significance was set at *p* < 0.05.

## Results

### Patients’ demographics

Routine follow-ups were conducted for all 54 patients included in the study up to 12 weeks postoperatively, and the data were not lost during the follow-up for any patient. No statistically significant differences were observed in patient demographics or preoperative blood test results. In addition, preoperative knee function, QoL, and pain scores in the three groups were comparable and not statistically different (Table 1).

**Table 1:** Baseline characteristics of the three groups

Variable	single-dose	two-dose	multiple-dose	*P* value (between group)
Patient characteristics				
Age (yr)	54.63 ± 6.71	57.81 ± 5.53	55.15 ± 6.17	0.250^a^
Gender(male/female)	6/12	5/13	8/10	0.566^b^
BMI	25.73 ± 2.41	26.92 ± 2.54	26.56 ± 1.94	0.166^a^
Medical history				
Diabetes	1	1	1	0.864^b^
Hypertension	3	1	2	
Arrhythmia	1	2	2	
ASA score				
I	10	11	13	0.842^b^
II	6	6	4	
III	2	1	1	
Preoperative blood tests				
Hb (g/L)	132.13 ± 11.01	129.32 ± 9.23	131.15 ± 10.33	0.714^a^
Hct (%)	40.16 ± 3.14	38.33 ± 2.75	39.14 ± 2.16	0.138 ^a^
D-dimer (mg/L)	0.23 ± 0.10	0.25 ± 0.10	0.17 ± 0.10	0.053 ^a^
FDP (mg/L)	2.66 ± 0.61	2.43 ± 0.59	2.58 ± 0.42	0.446^a^
LKS scoers	43.15 ± 7.76	42.60 ± 6.95	44.20 ± 7.27	0.802 ^a^
VAS scoers	5.12 ± 1.65	5.43 ± 1.14	5.38 ± 1.32	0.773 ^a^
QoL of SF-12				
PCS	32.62 ± 3.45	33.14 ± 4.16	32.73 ± 4.35	0.919 ^a^
MCS	49.73 ± 6.95	47.56 ± 6.18	50.14 ± 7.27	0.480 ^a^

*Abbreviations*: *ANOVA* analysis of variance, *BMI* body mass index, *ASA* American Society of Anesthesiologists, *Hb* haemoglobin, *Hct* haematocrit, *FDP* fibrinogen degradation products, *QoL* quality of life, *SF-12* 12-item Short Form Health Survey, *PCS* physical component summary scores, *MCS* mental component summary scores

Intergroup comparisons performed using ANOVA or Chi-square test (^a^ANOVA; ^b^Chi-square test)

### Primary outcomes

The significant differences were observed in the TBL among the three groups (*p* < 0.001). Statistically significant differences were observed between single-dose and two-dose (*p* = 0.022), single-dose and multiple-dose (*p* < 0.001), and two-dose and multiple-dose (*p* = 0.047) groups via pairwise comparison and statistical analysis. The same result was also demonstrated for the HBL in single-dose (561 ± 216 mL), two-dose (493 ± 165 mL), and multiple-dose (416 ± 141 mL) groups, with an intergroup *p-value* of < 0.001 (Table 2).

**Table 2:** Comparison of the primary and secondary outcomes of the three groups

Variable	single-dose	two-dose	multiple-dose	*P* value	Intergroup comparison		
					*P*_*1*_ *P*_*2*_ *P*_*3*_		
Primary outcomes							
TBL(mL)	1138 ± 404	978 ± 311	727 ± 278	0.002^a^	0.192	0.001	0.015
HBL(mL)	581 ± 216	493 ± 165	416 ± 141	< 0.001^a^	0.179	0.010	0.142
Transfusion rate (n,%)	3(16.7%)	1(5.6%)	1(5.6%)	0.414^b^	–	–	–
Secondary outcomes							
Duration of surgery (min)	137.23 ± 16.34	139.41 ± 17.55	125.83 ± 20.15	0.063^a^	–	–	–
Hospitalization days (d)	7.74 ± 1.20	7.95 ± 1.35	7.45 ± 1.00	0.456^a^	–	–	–
Drainage volume (mL)	446 ± 218	322 ± 185	274 ± 118	0.017^a^	0.074	0.006	0.360
Maximum Hb drop(g/L)	20.22 ± 12.31	19.84 ± 10.77	17.84 ± 10.77	0.795^a^	–	–	–
Postop. Laboratory values							
Hb (g/L)							
POD#1	113.17 ± 10.14	113.93 ± 9.74	115.15 ± 10.22	0.837^a^	–	–	–
POD#3	104.16 ± 12.33	106.71 ± 11.51	113.64 ± 10.18	0.043^a^	0.526	0.017	0.064
Hct (%)							
POD#1	34.46 ± 3.77	34.51 ± 2.35	36.14 ± 3.26	0.207^a^	–	–	–
POD#3	31.07 ± 5.13	31.37 ± 3.43	33.52 ± 2.87	0.135^a^	–	–	–
D-dimer (mg/L)							
POD#1	3.96 ± 1.14	4.01 ± 1.59	3.74 ± 1.33	0.820^a^	–	–	–
POD#3	2.62 ± 0.81	2.88 ± 0.72	2.33 ± 0.60	0.116^a^	–	–	–
FDP (mg/L)							
POD#1	7.67 ± 1.21	7.51 ± 1.39	6.84 ± 0.93	0.096^a^	–	–	–
POD#3	4.84 ± 1.16	4.64 ± 1.03	4.68 ± 0.87	0.827 ^a^	–	–	–

*Abbreviations*: *ANOVA* analysis of variance, *POD#1* the first postoperative day, *POD#3* the third postoperative day

*P*_*1*_ represents the p value obtained by comparison between single-dose and two-dose groups

*P*_*2*_ represents the p value obtained by comparison between single-dose and multiple-dose group

*P*_*3*_ represents the *p* value obtained by comparison between two-dose and multiple-dose group

Intergroup comparisons performed using ANOVA or Chi-square test (^a^ANOVA; ^b^Chi-square test)

Three patients, one patient, and one patient in the single-dose, two-dose, and multiple-dose groups, respectively, were transfused with two units of PRBCs owing to postoperative symptoms of anaemia. No statistically significant difference in the transfusion rate was observed among the three groups (*p* = 0.414).

### Secondary outcomes

#### Duration of surgery and hospitalisation

The mean values of the duration of surgery in the single-dose, two-dose, and multiple-dose groups were 137.23, 139.41, and 125.83 min, respectively, with no significant intergroup differences (*p* = 0.063). In addition, there was no statistically significant difference in the number of hospitalisation days among the three groups (*p* = 0.456) (Table 2).

#### Postoperative blood test results

**Fig. 1:**
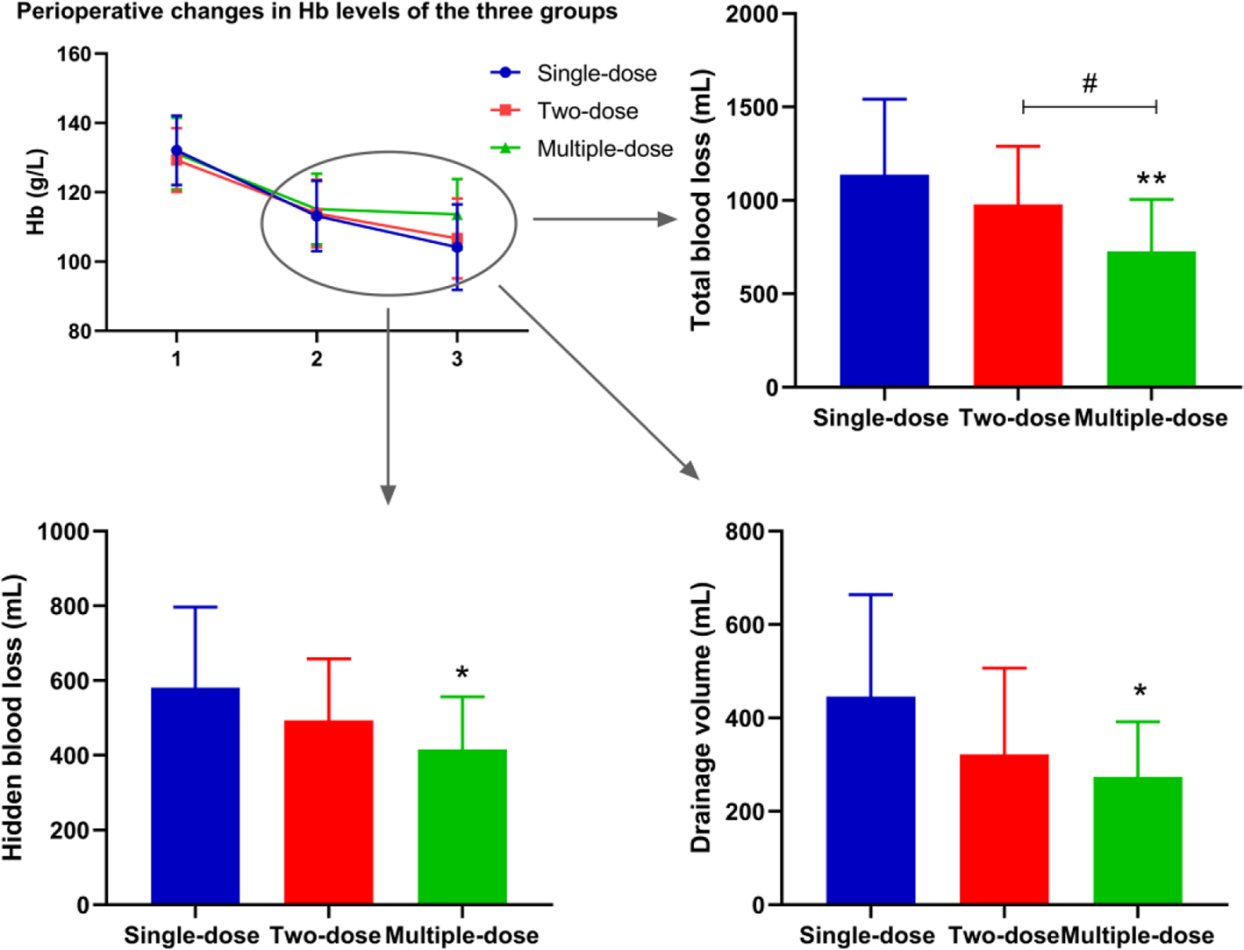
Changes in perioperative haemoglobin levels among the groups and the corresponding blood loss comparison. Intergroup comparisons were performed using ANOVA. ** indicated *p* < 0.01; *indicated *p* < 0.05 (Multiple-dose group VS. Single-dose group). # indicated *p* < 0.05 (Multiple-dose group VS. Two-dose group)

The results of routine blood tests and blood coagulation tests for the three groups are summarised in Table 2. In the single-dose, two-dose, and multiple-dose groups, the mean values of postoperative Hb (on POD#1) were 113.17 ± 10.14, 113.93 ± 9.74 and 115.15 ± 10.22 g/L, respectively, with no significant intergroup difference (*p* = 0.837); however, the mean values of Hb on POD#3 were 104.16 ± 12.23, 106.71 ± 11.51 and 113.64 ± 10.18 g/L, with significant differences among the three groups (*p* = 0.043). The maximum decrease in Hb levels in the multiple-dose (17.84 ± 10.73 g/L) and two-dose (19.84 ± 10.77 g/L) groups was lower than that in the single-dose group (20.22 ± 12.33 g/L), but the difference was not statistically significant. Furthermore, the mean D-dimer and fibrin degradation product values did not differ significantly on POD#1 and POD#3.Changes in perioperative haemoglobin levels among the groups and the corresponding TBL and HBL comparison were in Fig. 1.

#### Vascular events, wound complications, and adverse reactions

In this study, the venous plexus of the calf muscle was the most common site for DVT, followed by the popliteal vein. No severe complications such as PE, myocardial infarction, or cerebral infarction were observed. No significant differences in wound complications or adverse reactions were observed among the three groups. The associated data were listed in Table 3.

**Table 3:** Other complications including thromboembolic events, wound complications, and adverse reactions of TXA

Variable	single-dose	two-dose	multiple-dose	*P* value (between group)
Thromboembolic events				
DVT	1	0	1	0.595
PE	0	0	0	–
Incision-related complications				
Dehiscence	0	0	0	–
Hematoma	1	1	0	0.595
Edge necrosis	0	0	0	–
Infection	0	0	0	–
TXA adverse reactions				
Dizziness or headache	1	1	2	0.763
Nausea and Vomiting	1	2	2	0.802
Rash	0	0	1	0.361

*Abbreviations*: *TXA* tranexamic acid, *DVT* deep vein thrombosis, *PE* pulmonary embolism

Chi-square test for intra-group comparison

#### Knee function, QoL and pain assessment

The LKS scores of the three groups in the follow-up period were not statistically significant. The multiple-dose group had the advantage of controlling early postoperative pain; however, this advantage was not sufficient to exhibit a statistically significant difference (*p* = 0.099 and 0.459, respectively). The results demonstrated a significant difference in the physical component summary (PCS) scores among the three groups at 6- and 12-weeks postoperatively (*p* = 0.049 and 0.024, respectively). No differences in the mental component summary scores were observed among the three groups (Table 4).

**Table 4:** Outcomes for the three groups

Variable	single-dose	two-dose	multiple-dose	*P* value	Intergroup comparison		
					*P*_*1*_ *P*_*2*_ *P*_*3*_		
LKS scoers							
Postop. 6-week	69.70 ± 5.96	70.14 ± 7.32	70.67 ± 6.43	0.907	–	–	–
Postop. 12-week	70.42 ± 8.27	74.25 ± 10.14	73.67 ± 9.05	0.409	–	–	–
VAS scoers							
POD#2	5.13 ± 1.45	4.66 ± 0.97	4.27 ± 1.05	0.099	–	–	–
POD#4	4.15 ± 1.01	3.91 ± 0.89	3.74 ± 1.04	0.459	–	–	–
Postop. 6-week	2.26 ± 0.85	2.23 ± 0.95	2.17 ± 0.77	0.950	–	–	–
Postop. 12-week	1.73 ± 0.43	1.75 ± 0.50	1.68 ± 0.55	0.909	–	–	–
SF-12 QoL							
PCS							
Postop. 6-week	39.17 ± 4.24	40.64 ± 3.76	43.03 ± 5.65	0.049	0.279	0.027	0.144
Postop.12-week	43.09 ± 5.97	45.95 ± 7.12	49.02 ± 6.75	0.024	0.200	0.004	0.161
MCS							
Postop. 6-week	51.86 ± 5.96	50.51 ± 7.01	53.63 ± 5.25	0.361	–	–	–
Postop.12-week	56.30 ± 7.76	56.69 ± 6.94	56.77 ± 6.87	0.978	–	–	–

*P*_*1*_ represents the p value obtained by comparison between single-dose and two-dose groups

*P*_*2*_ represents the p value obtained by comparison between single-dose and multiple-dose group

*P*_*3*_ represents the *p* value obtained by comparison between two-dose and multiple-dose group

Chi-square test for intra-group comparison

## Discussion

The advantages of TXA administration in unilateral opening-wedge HTO (OW-HTO) have been proven in many retrospective studies and meta-analyses [19–23]. Therefore, further reduction of the TBL, HBL, and drainage volume after SBDTT-HTO has been an important directive. The purpose of this study was to determine whether the sequential IV TXA regimen is effective and safe, from the perspective of reducing perioperative blood loss and drainage volume, with an additional detailed assessment.

The primary outcomes obtained in this study indicate that sequential IV TXA can effectively reduce TBL, HBL,drainage volume and maintain postoperative Hb values in patients. Compared with the TBL observed with a single-dose of TXA, two-dose reduced TBL by approximately 160 mL and multiple-dose reduced TBL by approximately 311 mL (1138 vs. 827 mL, *p* = 0.011). The calculation of HBL also confirmed the advantages of multiple doses of TXA. The mean HBL volumes in the multiple-dose, two-dose and single-dose groups were 416, 493, and 561 mL, respectively (*p* < 0.001). The changes in postoperative drainage volume among the three groups showed a similar trend to those in TBL and HBL. The drainage volume was reduced by approximately 124 and 172 mL in the two-dose and multiple-dose groups compared with that in the single-dose group. On POD#3, the Hb level in the multiple-dose group was higher than that in the two-dose and single-dose groups (*p* < 0.05), and the maximum Hb drop was also the lowest, indicating that multiple doses of TXA can reduce blood loss from POD#1 to POD#3. In particular, patients in multiple-dose had more advantages during the postoperative rehabilitation period, including early pain reduction and better QoL, without an increase in the incidence of postoperative vascular events, incision complications, and adverse reactions.

The effect of TXA administration on blood loss and subsequent complications after major orthopaedic surgery has been well described in previous studies. As a traditional anti-fibrinolytic drug, the effectiveness of TXA when administered intravenously, topically, or in combination has been proven. Aggarwal et al. [24] found that topical administration of 15 mg/kg TXA in simultaneous bilateral TKA can effectively reduce TBL during the perioperative period, and the Western Ontario and McMaster Universities Arthritis Index score at 12 weeks and 6 months was better than that of the IV administration group. Kim et al. [23] injected TXA intravenously at a dose of 10 mg/kg before and 6 h after tourniquet application and 24 h after surgery. The results demonstrated that the Hb level in the TXA group was higher than that in the control group on POD#1, POD#2, and POD#5 (*p* < 0.001). Moreover, the total drainage volume and TBL were lower in the TXA group than in the control group (*p* < 0.001). To the best of our knowledge, the pharmacokinetic study of IV TXA demonstrated that its half-life is approximately 3 h, and the therapeutic plasma concentration of TXA is 10 h from the time of administration [25]; however, the hyper-fibrinolytic state of the body, which is caused by surgical trauma, reaches its peak at 6 h after surgery and continues until 18–24 h [26, 27]. The current trial had a shorter IV administration gap than that in the study mentioned above. Therefore, our study had a theoretical advantage in suppressing early postoperative fibrinolysis, thereby reducing postoperative blood loss and drainage volume. Our results confirm the superiority of the multiple-dose IV TXA treatment regimen to the two-dose and single-dose regimens. Some scholars reached an optimistic conclusion regarding the advantages of multi-dose IV TXA for TKA without tourniquet application [26] and observed that the use of multiple-dose IV TXA did not increase blood loss during surgery.

Thus, good perioperative blood management can reduce blood loss and transfusion due to surgical trauma, reduce the incidence of anaemia, and maintain a high postoperative Hb level. It is also closely related to postoperative rehabilitation of physical function. Anti-fibrinolytic therapy, an important aspect of blood management that is considered to be closely related to the concept of enhanced recovery after surgery, has emerged as an area of particular research focus, and TXA has been apply this strategy in a clinical setting in our institution [28–31].

The safety of TXA during the perioperative period of major orthopaedic surgery has been controversial. Hence, the pace of clinical promotion of TXA has been reduced [32]. More safety concerns are associated with DVT and PE than with cerebral infarctions and gastrointestinal bleeding. Although some scholars reported that the incidence of DVT and PE in Asians is significantly lower than that in Europeans and Americans, the incidence is still higher following major surgery of lower extremity orthopaedics [33]. Hence, this problem deserves attention. At present, most orthopaedic clinical trials are designed to test the haemostatic effect of TXA instead of its safety. For rare complications such as PE, the current clinical trial could not reach a definitive conclusion owing to the sample size. However, a large-scale retrospective study from China (including 1907 THA and 1505 TKA cases) concluded that TXA reduced blood transfusion rates without increasing the prevalence of DVT/PE [34]. In this study, we used a combination of mechanical compression devices and chemical drugs to prevent thromboembolic events, and the results obtained were consistent with those of the above study. Therefore, we suggest that with reasonable intervention after SBDTT-HTO, the administration of multiple doses of IV TXA does not increase the risk of thrombotic events (*p* > 0.05).

In this study, there was one incision-site haematoma case in the single-dose and two-dose groups. In general, measures to avoid subcutaneous hematoma include appropriate soft tissue handling, meticulous haemostasis, wound closure without excess tension, and regular postoperative care. During the surgical procedure, the plate is placed in the subcutaneous plane and is covered only by a very thin layer of fascia and the skin; hematoma caused by exudation from the osteotomy site may endanger wound healing [22]. In addition, a study showed that reducing the perioperative blood transfusion rate can reduce the incidence of wound complications [35]. To our knowledge, although our study was the largest study on TXA administration in patients undergoing SBDTT-HTO, the sample size was still too small to detect differences in these rare complications. The incidence of adverse reactions of TXA, especially neurological symptoms, was higher in the multiple-dose group then single-dose and two-dose group. However, we could not determine whether this was because of adverse reactions after general anaesthesia or IV TXA. Notably, in the multiple-dose group, one patient developed rashes after being administered IV TXA; however, the symptoms improved after administration of dexamethasone.

Tzatzairis et al. [26] observed that three doses of IV TXA in TKA can achieve higher knee function and QoL and significantly reduce pain in the early and late postoperative periods. Palanisamy et al. [22] observed that after OW-HTO, the VAS score of the TXA group was lower than that of the control group on POD#2 but had no clinical significance because the difference was minor. Li et al. [19] observed that the combined IV and local TXA protocol did not effectively improve knee HSS and VAS scores in patients after surgery (at 6 months postoperatively). A recent study has demonstrated that the use of a drainage tube did not increase blood loss when TXA was administered intravenously, and the VAS score and calf swelling in the early postoperative period were lower than those in the control group, effectively reducing the incidence of incision complications [8]. Interestingly, the multiple-dose TXA regimen can effectively alleviate early postoperative pain in patients and can help patients exercise knee joint mobility as soon as possible. Early knee function recovery and pain improvement are favourable factors for predicting the QoL after HTO. This is the reason for the higher SF-12 PCS score in the three-dose group since the early postoperative period.

This study had certain limitations. First, the sample size of this study was small, and the study was conducted at a single centre. Prospective, large-scale, randomised, case–control studies are required to confirm these findings. Second, according to the perioperative rehabilitation guidelines for major orthopaedic surgery established by our institution, all patients received preventive anticoagulation after surgery, which might have had an impact on postoperative blood loss. Third, given the effectiveness and safety of TXA in previous studies, patients were not recruited to the control group. Fourth, some recent studies have highlighted surgery-associated factors that are associated with perioperative period blood loss during HTO, especially tourniquet application and navigation. Several studies have reported advantages associated with not using tourniquets in HTO, and the use of navigation to reduce blood loss. Here, we realized the advantages of navigation and the disadvantages of tourniquet application, and subsequent research will focus on performing navigation SBDTT-HTO without a tourniquet.

## Conclusions

For SBDTT-HTO, sequential IV TXA reduced blood loss with no apparent increase in the incidence of complications. In addition, it more effectively maintained the postoperative Hb value, which is conducive to pain reduction, functional recovery, and a higher QoL score in the early postoperative period.

## Data Availability

All data generated or analysed during this study are included in this published article.

## Abbreviations

TXA: Tranexamic acid
IV: Intravenous
KOA: Knee osteoarthritis
HTO: High tibial osteotomy
CW-HTO: Closing-wedge HTO
OW-HTO: Opening-wedge HTO
QoL: Quality of life
DVT: Deep vein thrombosis
Hb: Haemoglobin
Hct: Haematocrit
VAS: Visual analogue scale
PE: Pulmonary embolism
TBL: Total blood loss
HBL: Hidden blood loss
PBV: Preoperative blood volume
LKS: Lysholm Knee Score
PCS: Physical component summary
TKA: Total knee arthroplasty
